# Selection of Male Donors in Local Chicken Breeds to Implement the Italian Semen Cryobank: Variability in Semen Quality, Freezability and Fertility

**DOI:** 10.3390/vetsci11040148

**Published:** 2024-03-27

**Authors:** Manuela Madeddu, Luisa Zaniboni, Stefano Paolo Marelli, Cristina Tognoli, Silvia Belcredito, Nicolaia Iaffaldano, Michele Di Iorio, Silvia Cerolini

**Affiliations:** 1 Department of Veterinary Medicine and Animal Science, University of Milan, via dell’Università 6, 26900 Lodi, Italy; manuela.madeddu@unimi.it (M.M.); stefano.marelli@unimi.it (S.P.M.); cristina.tognoli@unimi.it (C.T.); silvia.belcredito@unimi.it (S.B.); silvia.cerolini@unimi.it (S.C.); 2 Department of Agricultural, Environmental and Food Science, University of Molise, 86100 Campobasso, Italy; nicolaia@unimol.it (N.I.); michele.diiorio@unimol.it (M.D.I.)

**Keywords:** local chicken breeds, semen cryopreservation, semen cryobank, artificial insemination, fertility, embryo viability

## Abstract

**Simple Summary:**

The conservation of local chicken breeds is globally considered because of their interesting characteristics, such as their tolerance to heat stress, resistance to diseases and capacity to survive in severe environments. In recent years, conservation programs of native chicken breeds have been developed in Italy, and this work has focused on *Bionda Piemontese*, *Bianca di Saluzzo* and *Pepoi*, the first two of which are catalogued as endangered and the third as a ‘critically maintained’ breed. Semen cryopreservation is the only method that is currently feasible for the ex situ management of genetic diversity in birds, and poultry sperm shows a greater susceptibility during the freezing/thawing process due to its unique features. Therefore, the choice of the most suitable rooster sperm donor is fundamental in the establishment of a semen cryobank. The present study aims to analyze the efficiency of bird and semen management required for the implementation of the Italian Semen Cryobank of Autochthonous Chicken and Turkey Breeds created with the “TuBAvI” project. Our results suggest that male donors need to be selected based on the quality of fresh ejaculates, regardless of sperm outcome after freezing/thawing, in order to identify individuals who can ensure the survival of a breed with regard to increased fertilizing capacity.

**Abstract:**

Native breed conservation is an important component of poultry biodiversity. The aim of this work is to describe different steps that lead to donor selection for the implementation of the Italian Semen Cryobank of Autochthonous Chicken and Turkey Breeds. The variability within and between breeds was evaluated, and the stored semen reproductive capacity was in vivo tested using artificial insemination. Semen from *Bionda Piemontese*, *Bianca di Saluzzo* and *Pepoi* roosters was collected and processed. Concentration, volume, sperm membrane integrity, total motile sperm, progressive motile sperm and kinetic parameters were analyzed; sperm parameters accounting for bird variability were used to select male donors. Fresh semen quality parameters measured in donor ejaculates showed significant differences between breeds; no differences were found after cryopreservation. Variability in the fertilizing ability of cryopreserved semen was found within a breed (5–16%) and between birds within a breed (BP = 3–7%; BS = 7–31%; PP = 6–22%); only sperm quality parameters measured in fresh ejaculates, not frozen/thawed, may be associated with in vivo fertility results. In conclusion, sperm concentration and progressive motility were successfully used as selection parameters to identify chicken male donors with improved sperm quality for sperm cryobanking. However, new reliable sperm markers to predict cryopreserved semen’s fertilizing ability are required.

## 1. Introduction

The concern for saving biodiversity has been rapidly growing in recent decades, and it has become a primary target worldwide. Based on the Food and Agriculture Organization [1], 48% of indigenous breeds of domesticated and farmed animals are at risk of extinction in the Caucasus and Europe. In birds, about 50% of poultry breeds are classified as extinct, critical or endangered [2], and the proportion of unknown risk status populations is greater in birds (61%) compared to mammals (51%) [3]. In Italy, a literature survey reported 90 Italian breeds of different avian species in 2001, represented by chicken breeds as the majority (*n* = 53); however, 61% were classified as extinct, and only 9% were not at risk [4]. Recent data confirmed the presence of twenty-two chickens and eight indigenous turkey breeds in rural farming systems and the low bird population, corresponding to a risk status in the majority of them [5]. Over the last years, conservation programs on Italian chicken breeds implementing the in situ technique have been developed at the local level in several regions: Emilia Romagna [6,7,8,9], Lombardy [10,11,12] and Tuscany [13]. The Italian National Registry (INR) of local poultry breeds was established in 2014 as part of a large cross-sectional conservation project directed by the Italian Ministry of Agricultural, Food and Forestry Policies [14], with the aim of providing national guidelines for the safeguard and productive valorization of native breeds. The INR includes a list of chicken (*n* = 22), turkey (*n* = 8), guinea fowl (*n* = 7), duck (*n* = 4), goose (*n* = 3) and pigeon (*n* = 8) breeds; certified standards for each breed; and guidelines for breeding management [15]. 

The long-term maintenance of live populations is usually the first effort to prevent biodiversity losses; however, small populations undergo risks related to fluctuations in size, inbreeding, disease outbreaks and environmental disasters. Therefore, advanced and broad programs for the conservation of animal genetic resources are recommended to associate the priority in situ technique with the complementary ex situ in vitro technique in order to improve conservation potential and prevent the risk of dramatic or even total loss in biodiversity [16]. The implementation of the ex situ in vitro technique is considered essential to halt the current drain in biodiversity and rescue extant resources in the poultry sector, including commercial lines, indigenous breeds and experimental lines [17]. 

Cryopreservation allows the storage of biological material without degeneration for at least thousands of years [18] and presumably much longer. This material can be used to insert genetic diversity into in vivo populations, decreasing inbreeding levels and increasing breed diversity in the case of a genetic bottleneck. Therefore, the association of live animals with cryopreserved germplasm can be a strong tool in conservation [19]. Sperm cryobanking is the most common method for the in vitro conservation of genetic resources in endangered species, including both wild and domestic animals. In particular, in birds, sperm cryopreservation is the feasible biotechnology implemented for the ex situ conservation of genetic resources, and alternative methods exist in the form of primordial germ cells. The cryopreservation of germplasm in birds is a peculiar challenge due to their unique biological features, which include the macrolecithal egg, the hen being the heterogametic sex, the presence of sperm storage tubules in the hen’s oviduct and the morphology of sperm, resulting in high cellular sensitivity relative to the cryopreservation process [20]. 

In order to plan sperm cryobanking, FAO [21] suggests a minimum number of 25 donors and some important principles, such as conserving small germplasm quantities from many donor animals rather than large quantities from few donors and storing the breeds as pure lines rather than gene pools. If the at-risk breed is generally dispersed geographically, the possibility of collecting from multiple animals is restricted; therefore, the collection of samples from the recommended number of animals will be difficult, and a longer period of sampling will be required. According to FAO cryoconservation guidelines [22], three to ten males should be selected each year in the case of a small population, at least 20 doses of semen from each male should be stored, and an increasing number should be considered based on the reproductive capacity of the species (low capacity requires more doses) and the population size (larger population requires more doses). For any breeding program, independent of the population size, the recurrent cryogenic storage of genetic material is suggested as a backup in case (genetic) problems occur. Furthermore, when selecting male donors, priority should be given to individuals who are known to produce semen that is of good quality after it is frozen and thawed [22]. 

Artificial insemination (AI) centers exist in many countries for mammalian species (i.e., cattle, small ruminants, horses, pigs), providing facilities and expertise that may facilitate the collection, processing, storage and use of semen for cryobanking. In contrast, in poultry species, despite AI being considered a reproductive technique that is valuable for the poultry industry in maximizing male efficiency [23,24], AI is extensively practiced only in commercial turkeys and occasionally used in commercial chickens, mainly for pedigree lines [25]. Moreover, when used, AI requires very efficient technical and economic management, it is usually performed on farms, and pooled semen is used in order to avoid the risk of individual sub-fertility [26]. The advent of sperm cryobanks for the conservation of poultry genetic resources highlighted the need to build up knowledge, which is so far lacking in variability in semen production and quality within and between breeds/lines in order to set up successful procedures for the selection of semen donors.

The present study aims to analyze the efficiency of bird and semen management required for the implementation of the Italian semen cryobank of autochthonous chicken and turkey breeds [27]. The selection criteria for identifying donor males, semen quality before and after cryopreservation and the in vivo fertility of cryopreserved ejaculates were studied in three Italian chicken breeds in order to set up guidelines to optimize bird and semen management for the implementation of sperm cryobanking in rare chicken breeds. The expected results will be also a useful model for other avian species. 

**Figure 1:**
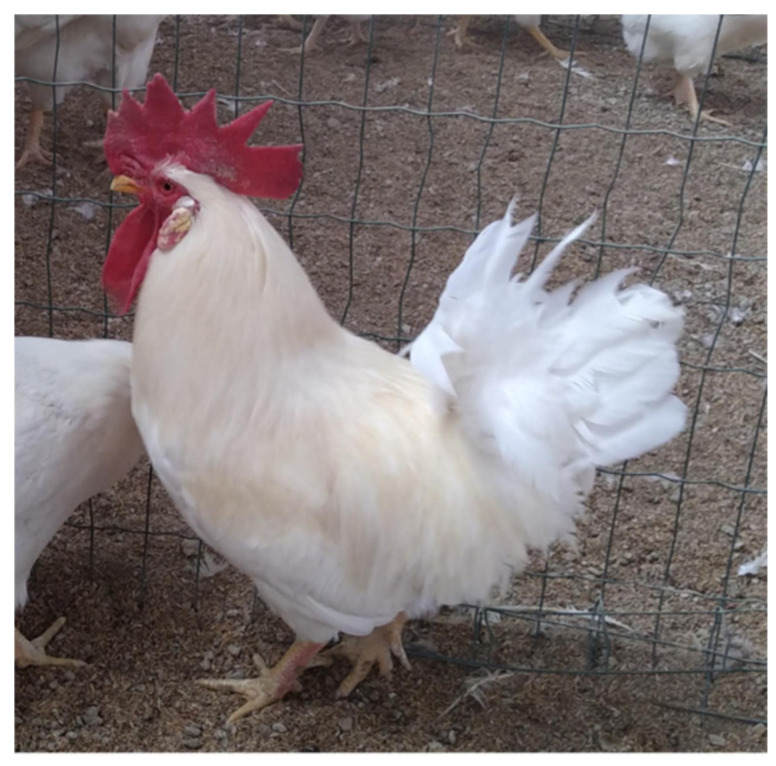
*Bianca di Saluzzo*.

**Figure 2:**
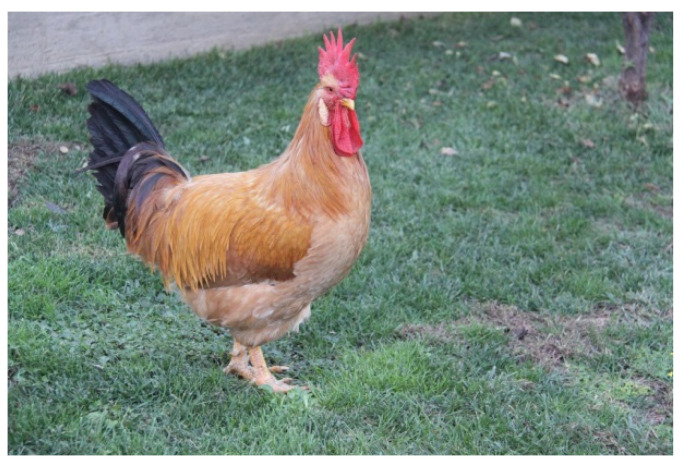
*Bionda Piemontese*.

**Figure 3:**
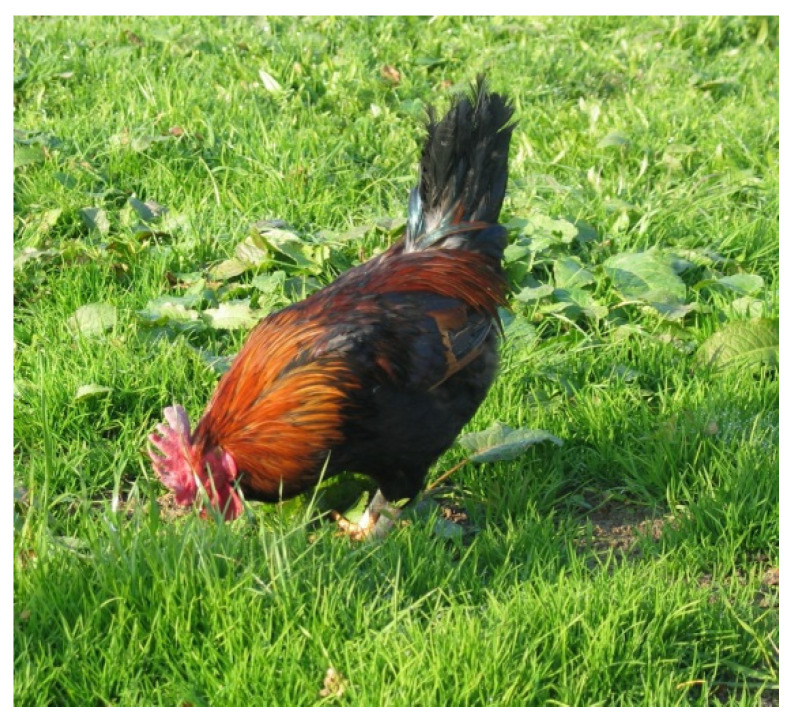
*Pepoi*.

The three chicken breeds chosen for this study are considered at risk and are as follows: *Bianca di Saluzzo* (BS) (Figure 1), *Bionda Piemontese* (BP) (Figure 2) and *Pepoi* (PP) (Figure 3). All breeds are considered dual purpose; BS and BP are from the Piemonte region in northwest Italy, and PP is from the Veneto region. The population sizes were 286, 566 and 899 birds with respect to BS, BP [1] and PP [5] breeds, respectively.

## 2. Materials and Methods

### 2.1. Bird Management

Roosters of three Italian breeds were used: *Bianca di Saluzzo* (*n* = 18; BS), *Bionda Piemontese* (*n* = 19; BP) and *Pepoi* (*n* = 19; PP). Roosters were housed at 20 weeks of age in individual cages at the Poultry Unit, Animal Production Centre, University of Milan (Lodi, Italy). The birds were kept in a controlled environment at 20 °C and a 14L:10D photoperiod. Birds were fed ad libitum with a standard commercial chicken breeder diet (2800 kcal ME/kg, 15% crude protein) and drinking water. Bird handling conformed to the principles presented in the Guidelines for the Care and Use of Agricultural Animals in Research and Teaching [28]. The Animal Welfare Committee of the University of Milan evaluated and authorized the experimental protocol and bird management (OPBA_43_2022). 

### 2.2. Semen Collection

Semen collection was routinely performed twice weekly after two weeks of training. Semen was collected according to the dorso–abdominal massage technique initially described by Burrows and Quinn [29]. In brief, the bird’s back and ventral–abdominal region were massaged; then, the cloaca was everted and gently squeezed to cause ejaculation, and semen was collected into a glass tube connected to an electric aspirator. The frequency and quality of ejaculates were recorded. Individual body weight was recorded.

### 2.3. Sperm Processing

On each day of collection, individual ejaculates were collected into graduated tubes, and the volume (vol, mL) was recorded. Sperm concentrations (conc, ×10^9^/mL) were calculated using a calibrated photometer (Acucell Photometer, IMV Technologies, L’Aigle, France) at a wavelength of 535 nm [30]. Total sperm output (TSO: vol × conc × 10^9^) was determined. Ejaculates were then diluted to 1.5 × 10^9^ sperm/mL in a modified Lake pre-freezing extender, added with 0.1 M trehalose (LPF-T) [31], cooled at 5 °C and moved to the laboratory for further processing.

### 2.4. Sperm Motility and Kinetic Parameters Analysis

Sperm motility and kinetic parameters were measured in ejaculates using the Sperm Class Analyzer (SCA) software (version 4.0, Microptic S.L., Barcelona, Spain). A small aliquot (50 μL) of fresh ejaculates was further diluted in refrigerated 0.9% NaCl to 50 × 10^6^ sperm/mL, incubated for 20 min at room temperature and motility assessed at room temperature placing 10 μL of semen on a Makler chamber (Sefi Medical Instruments, Haifa, Israel) under a microscope. The following motility parameters were recorded: total motile sperm (TMS), progressive motile sperm (PMS) and some kinetic parameters (VCL, VSL, VAP, ALH, BCF, LIN, STR and WOB), previously described in detail by Mosca et al. [31]. In each sample, a minimum of three fields and 500 sperm tracks at 100× magnification were analyzed; 25 frames per second (Hz) and 25 frames per field were set. The following software settings were used: range cell size from 5 to 190 µm^2^; sperm classified as motile if VCL ≥ 13 µm/s; sperm classified as progressive if STR ≥ 70%.

### 2.5. Sperm Membrane Integrity

Sperm membrane integrity (SMI) was assessed using the two fluorescent dyes SYBR-14/propidium iodide (LIVE/DEAD Sperm Viability Kit, Molecular Probes^®^, Invitrogen, Carlsbad, CA, USA), as described by Rosato and Iaffaldano [32], but with minor modifications [31,33]. Sperm cells (*n* = 200) were evaluated in duplicate aliquots for every sample using a Zeiss (Axioskop 40-AxioCamICc 1) microscope and FITC filter fluorescence at 100× total magnification. 

### 2.6. Semen Cryopreservation Processing

After cooling at 5 °C for 20 min, semen was further diluted to 1 × 10^9^ sperm/mL in LPF-T, with final concentrations of 2% (*v*:*v*) N-Methylacetamide (NMA) added [34]. After equilibration at 5 °C for 1 min, semen was loaded into 0.25 mL French straws (IMV Technologies, L’Aigle, France) and frozen for 10 min over a nitrogen bath at 3 cm of height [35]. Different straw colors were used for each breeder. Straws were stored in a cryotank for at least 7 days before thawing. Ejaculates were collected and processed for several days until the required number of straws was stored. The straws were thawed at 5 °C for 100 s, and sperm quality was evaluated as previously described in fresh semen, with the exception of sperm motility, which was assessed immediately after thawing. 

### 2.7. Fertility Trial 

The donors with the highest number of semen doses were chosen for the in vivo trial. Frozen/thawed ejaculates from three roosters per breed (*Bionda Piemontese*: BP1, BP4 and BP17; *Bianca di Saluzzo*: BS28, BS35 and BS36; *Pepoi*: PP10, PP19 and PP20) were used for AI. Eighty-six hens (Tetra-SL egg-laying strain) were housed in cages at 19 weeks of age at the Poultry Unit and raised conforming to standard management guidelines for egg-laying hens. The photoperiod applied was 15L:9D photoperiod (light on 2:30 a.m.), and all inseminations were carried out between 2:30 and 4:30 p.m. using the method of Burrows and Quinn [36]. AI was performed after the oviposition rate reached 90%. The hens were randomly divided into nine groups (*n* = 9), each corresponding to a rooster (BP1, BP4, BP17, BS28, BS35, BS36, PP10, PP19 and PP20), and a further group (*n* = 5) was used as a control. Each hen was inseminated twice within one day of each other: days 1 and 3. The concentration of the insemination dose was 250 × 10^6^ sperm/hen, corresponding to 1 straw. Eggs were collected daily, from the 2nd to the 10th day after the first insemination (day 1), and they were set every 3 days. Fertility and dead embryos were identified via candling after 7 days of incubation. All clear eggs were opened to differentiate unfertilized germinal discs and very early embryonic death within 48 h of incubation. Fertility (%) was calculated with respect to the total number of eggs set, and embryo viability (%) was assessed with respect to the total number of fertilized eggs.

### 2.8. Statistical Analysis 

Values of descriptive statistics were calculated with respect to sperm quality traits recorded in the fresh ejaculates of all birds: mean, min, max and coefficient of variation (CV). In order to rank the birds within breed according to their semen production, an analysis of variance was performed on the breed dataset using the GLM procedure of SAS [37] to assess the effect of the bird as a source of variation. Student’s *t*-test was used to compare LSMeans, and statistical significance was set at *p* < 0.05. Analysis of variance regarding sperm quality traits recorded in fresh and frozen/thawed semen samples of donors selected for cryobanking was performed using the GLM procedure of SAS [37], and the breed was the only source of variation included in the model. Student’s *t*-test was used to compare LSMeans. Principal component analysis (PCA) was conducted on sperm parameters as variables. A scatterplot was produced [38]. Prior to statistical analysis, all percentage data were normalized with an arcsine transformation. Data are presented as LSMean ± SE. The chi-square test [37] was applied to fertility data in order to assess the effect of the following categories: breed and birds within breed.

## 3. Results

### 3.1. Semen Quality

The ejaculation of semen was successful in 78.9% (*n* = 15) of males in PP and BP breeds, while it was successful in 100% (*n* = 18) of males in the BS breed. The mean body weights were 1957, 2960 and 3283 g in PP, BS and BP birds, respectively. Very high variability was found in semen production and quality, and descriptive statistic parameters are reported per breed in Table 1. TSO was the most variable parameter in all breeds, and it strongly affected the amount of ejaculate available for further in vitro processing. PP birds ejaculated larger amounts of sperm, corresponding to 1.57 × 10^9^ TSO, and they also showed the lowest CV in sperm volume, concentration and TSO. The proportion of motile sperm and, in particular, of progressive motile sperm showed higher CV compared to the proportion of sperm with intact membrane (SMI). A high CV, above 30%, was also found in kinetic parameters VCL, VSL and VAP, directly related to the quality of progressive movement, whereas a lower CV was shown in the velocity ratio LIN and STR, the former varying from 15% to 21% and the latter from 9% to 13% according to the breed.

The results of the analysis of variance on the breed dataset showed a significant effect of the bird on quantitative semen parameters in all breeds: volume (*p* < 0.001), concentration (*p* < 0.05) and TSO (*p* < 0.001). In contrast, no significant bird effect was observed in qualitative semen parameters in all breeds, with the exception of PMS (*p* < 0.05). Therefore, the significant parameters of semen concentration and PMS were used as criteria to select male donors for cryobanking. Males that routinely provided ejaculates with at least 2 × 10^9^ mL/ concentration and a PMS > 10% were selected for cryobanking, represented by six males out of eighteen (33%) and nineteen (32%) in the BS and BP breeds, respectively, and seven males out of nineteen (37%) in the PP breed. 

Semen production and quality were greatly improved in semen donors compared to the initial population. Analysis of variance showed a significant effect of the breed on several sperm traits; LSMeans and SE per breed and *p* values are reported in Table 2. The PP breed was confirmed to provide ejaculates with significantly higher sperm concentrations, and then TSO was also significantly improved to 1.9 × 10^9^, whereas BP and BS breeds showed a very similar TSO, corresponding to 1.3 × 10^9^. SMI, PMS, LIN, STR, WOB and BCF mean values were significantly higher, and in contrast, VCL and ALH were significantly lower in PP ejaculates compared to BS and BP ejaculates (Table 2). Therefore, the PP breed was characterized by generally higher semen quality compared to the BP and BS breeds, exhibiting similar semen quality parameters. 

Ejaculates collected in several days (at least eight) from each male were frozen and analyzed. The LSMeans and SE of sperm quality traits recorded in semen samples after freezing/thawing and the *p* value of the breed factor are reported in Table 3. As expected, a generally significant decrease in sperm quality occurred after the freezing–thawing process irrespective of the breed. The results of the analysis of variance showed no significant effect of the breed on sperm quality after cryopreservation, with the only exception of ALH (*p* < 0.001). A generally relevant decrease was observed in SMI, TMS, PMS, VCL, VSL and VAP mean values after cryopreservation, and as a consequence, the decrease in the velocity ratios LIN, STR and WOB was less evident. The ALH mean value progressively increased from PP to BS to BP in cryopreserved ejaculates, and a significant difference was found between PP and BP samples (Table 3). The mean BCF values in frozen/thawed samples of all breeds were similar (Table 3). 

**Figure 4:**
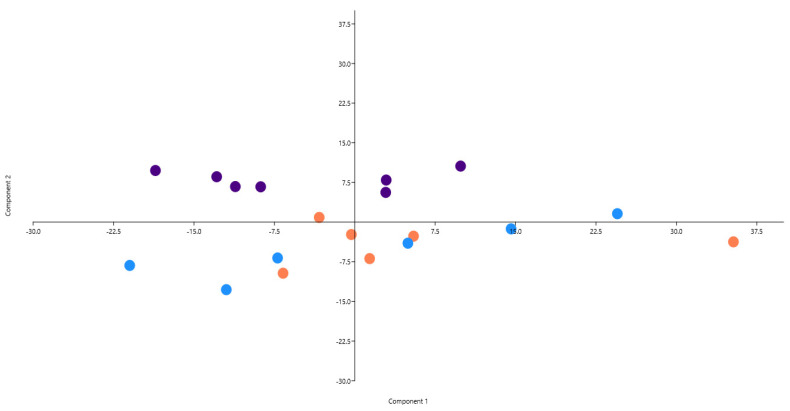
Scatterplot of principal component analysis (PCA) with the three local chicken breeds. Every sign represents a sample, and every color represents a breed (turquoise = *Bianca di Saluzzo*; orange = *Bionda Piemontese*; violet = *Pepoi*).

Principal component analysis (PCA) was carried out on sperm quality traits recorded in fresh and frozen/thawed semen samples of the donors selected for cryobanking in order to identify the more influential sperm variables, and a scatterplot was produced (Figure 4).

PCA provides objective information on the effects of the breeds on the analyzed samples. Principal components 1 and 2 define 90.26% of the variance of the dataset. PC1 is strongly linked to VCL and VAP in fresh semen: 0.76 and 0.45, respectively. PC2 is mainly influenced by SMI (0.48) and PMS in fresh semen (0.47). Scatter plot sample distribution underlines the clustering ability of the PP samples, whereas BS and BP show overlapping distributions in quadrants 3 and 4. The samples’ variance and grouping ability are defined on the PC2 axis (Figure 1). Interestingly, sperm variables recorded only in fresh and not cryopreserved semen account for breed variability. PCA results are strictly consistent with the results of the analysis of variance performed in the fresh (Table 2) and frozen/thawed (Table 3) semen of male donors.

### 3.2. Fertility and Embryo Viability

A total of 694 eggs were collected and set; only 114 eggs were fertile, equivalent to 16.43%. The proportion of live embryos after five days of incubation was 78% of total fertile eggs, and embryo mortality (22%) always happened within the first 48 h of incubation (Table 4). 

Fertility and live embryo data were significantly affected by the breed (chi-square test with *p* < 0.001). The distribution of fertile eggs and live embryos according to the breed is reported in Table 4. The AI of BS and PP cryopreserved semen provided 16% fertility and 81–79% embryo viability according to the breed. BP cryopreserved semen provided lower fertility and embryo viability, corresponding to 5% and 36%, respectively. As expected, the AI of fresh semen provided very high proportions in both fertility (100%) and embryo viability (87%), confirming the accuracy of egg conservation and the AI technique. Fertility and embryo viability were also significantly affected by the ‘bird within breed’ category (chi-square test with *p* < 0.001), and the results are reported in Table 5.

Very large bird variability was found for both parameters: fertility and embryo viability range values were 6–31% and 0–100%, respectively (Table 5). All BP donors showed very low fertility values compared to the overall mean value with a range between 2.7 and 6.9%; low fertility was also associated with the lack of viable embryos in two BP donors (BP1, BP17). Fertility was slightly improved in two BS donors (BS28 = 7.5% and BS36 = 8.45%) and significantly improved to 31% in the BS35 donor. Embryo viability was also improved in BS donors, reaching 57–95% with respect to range values. Fertility values of PP10 and PP20 donors were approximately 20%, associated with high embryo viability values (PP10 = 73%, PP20 = 100%), whereas significantly lower fertility was found in PP19, associated with lower embryo viability (25%). 

## 4. Discussion

Increasing our knowledge about intra-breed diversity in livestock species is important for the improvement of conservation programs for endangered populations [39,40,41]. Sperm cryobanking is still one of the most common tools for implementing the ex situ in vitro technique in conservation programs for many animal species, and the choice of donors plays a crucial role in the successful use of semen doses. 

In the present study, even if the large majority (at least 79%) of roosters in all breeds positively adapted to the handling for semen collection, variability in semen production was very high, and only a small number of birds (32–37%) were selected as donors. In order to create the French Avian Cryobank, a severe selection within a small population of 54 males of the Gauloise dorée breed was also reported, and of these, only 20 males (37% of the initial population) were selected as semen donors. Furthermore, only seven donors provided ejaculates that were useful in producing semen doses, whereas thirteen donors provided ejaculates that were then used in pooled samples for cryopreservation processing [42].

Large bird variability in fresh semen traits related to the number of ejaculates (volume, concentration and TSO), and PMS was a common feature in all breeds; in contrast, no bird variability within breed was observed with respect to sperm membrane integrity, total motility and kinetic parameters. The number of sperm ejaculated and their functions are related to physiological and technical factors; in fact, the efficiency of spermatogenesis within seminiferous tubules is mainly involved in association with the collection technique, the handling ability of the operator and the frequency of collection [26]. Variability in similar semen quality parameters between birds within breed/line was previously reported. A large range was found with respect to ejaculation volume, 0.05–1.0 mL, and concentration, 0.7–4.6 × 10^9^/mL, in the Castellana chicken breed [43]. Bird variability in ejaculate volume allowed to rank Thai native roosters from Pradu Hang Dam and Chee breeds into two groups characterized by high (0.35–0.36 mL) and low (0.22 mL) semen volume; the former group was also characterized by higher sperm concentration, progressive sperm movement and semen pH, whereas no difference in sperm viability was found [44]. Individual bird variability in volume, sperm concentration and motility were also observed in the fresh semen of capercaillie (*Tetrao urogallus* L.) males [45]. Sperm mobility data, corresponding to the penetration ability into a dense substrate (6% Accudenz solution), recorded in a large chicken population showed a normal distribution [46], and this quantitative trait was successfully used to categorize 27-week-old New Hampshire roosters. Sperm mobility was mainly related to the concentration of rapidly moving sperm, and a lower influence was associated with VSL [47]. 

A general improvement in semen quality was found in all breeds after the selection of male donors, even if a threshold was identified only for TSO and PMS. In the selected population, SMI mean values ranged from 91 to 95%, TMS from 89 to 92% and PMS from 24 to 30% according to the breed. Breed differences were found in several semen quality parameters, and PP birds provided ejaculates that were characterized by a higher TSO, which was associated with higher SMI, PMS, VCL, LIN, STR, WOB and BCF, compared to BS and BP birds providing ejaculates of similar lower quality. In particular, SMI and PMS in fresh semen were confirmed to be the most relevant semen traits in accounting for breed variability according to PCA results. The semen quality, mainly SMI and PMS, of BS and BP male donors recorded in the present study was higher compared to the mean values reported in the semen of the same breeds by Iaffaldano et al. [27]. Semen quality traits have been measured for the first time in the ejaculates of PP roosters and reported. Fresh semen quality in the PP breed, represented by SMI, TMS and PMS, was very similar to the fresh semen quality previously reported in the *Mericanel della Brianza* (MB) breed [27], even if low volume and concentrations were found in the latter breed. PP and MB birds are both characterized by small body size, being the only two Italian bantam breeds. Breed variability in semen volume, sperm concentration and morphology were reported in fancy chicken breeds such as White Crested Black Minorca, Greenleg Partridge, Italian Partridge and Black Minorca, with the prevalence of different breeds reflecting the semen trait [48]. Semen collected from Black Minorca roosters was also reported to have high plasma membrane integrity (91%), which is associated with 87% intact acrosome and 95% active mitochondria [49]. In contrast, similar high sperm viability (85–90%) and motility (80-90%) were found in the fresh semen of South African indigenous chicken breeds Ovambo and Potchefstroom Koekoek [50].

The freezing protocol used in this study was previously developed in different trials [31,34,35,36,51,52] and established as the official freezing method for the Italian Semen Cryobank of Autochthonous Chicken and Turkey Breeds [27]. As expected and already reported in commercial lines [34], semen quality in frozen/thawed semen samples was greatly reduced; SMI decreased from 91–95% to 31–38% and PMS decreased from 25–30% to almost 3%. The in vitro quality of frozen/thawed ejaculates was very similar in all breeds, with the only exception of ALH. The severe sperm cryodamage did not prevent in vivo fertilization, and all frozen/thawed ejaculates provided fertile eggs with variable results. Despite the similarity in conventional in vitro semen parameters, the AI of cryopreserved semen revealed a breed effect on fertility and embryo viability, with both mean values being very similar in BS (16% and 81%, respectively) and PP (16% and 79%, respectively) breeds and lower in the BP (5% and 36%, respectively) breed. Furthermore, the two breeds with similar mean values were characterized by different bird performances. In the PP breed, semen from two donors (out of three) provided similar fertility values above 20%, and this was also associated with high embryo viability above 73%; in contrast, in the BS breed, semen from one donor only (out of three) provided good fertility (31%), which was associated with high embryo viability (95%). The present results suggest a relation between the in vitro quality of fresh and not frozen/thawed semen and the fertilizing ability of cryopreserved sperm. In fact, PP birds provided ejaculates with improved sperm membrane integrity and motion parameters that were able to provide consistent in vivo fertility after cryopreservation processing. The same relation was lacking if semen quality was assessed after thawing and before AI; therefore, different sperm traits should be investigated to extend the characterization of sperm phenotypes in high-quality ejaculates.

Poultry semen freezability is conditioned by many factors, including variations among breeds and lines [53,54,55,56]. Using the same freezing protocol implemented in the present study, 9% fertility associated with 41% viable embryos was reported in a commercial egg-laying line [34]. Very variable fertility values were reported in different chicken breeds after the AI of cryopreserved semen in the presence of the cryoprotectant NMA: zero value in Nicobari fowl [57], 5% in free range lines [58], 35% and 57% in the Korean Oge breed [59,60], and more than 80% in the Yakido Japanese breed [61]. Large breed variability in fertility values, corresponding to a range of 29–83% [62,63,64], was also reported in semen samples cryopreserved in the presence of glycerol, which is considered the most effective cryoprotectant for chicken semen [65,66]. 

It is commonly accepted that one essential criterion for predicting the ability of sperm to resist freezing and thawing processing is the quality of fresh semen [43,67,68,69]. The predictive value of sperm in vitro markers to assess the fertilizing ability of cryopreserved semen is greatly relevant for the selection of male donors. Frozen/thawed ejaculates collected from 16 males of the same line expressed a wide range of fertility values from 6 to 94%. Sperm fluidity measured in fresh semen was the most predictive trait of the fertilizing ability of frozen/thawed semen, whereas the same sperm trait measured after freezing/thawing did not show a similar predictive value [67]. Furthermore, sperm fluidity followed by viability and then objective motility measured in fresh ejaculates accounted for 85% of the variability in the fertilizing ability of frozen/thawed sperm [67]. The lack of association between in vitro sperm quality, mainly sperm membrane integrity and motility, and the in vivo fertility of cryopreserved semen was also reported in a study that aimed to compare the cryoprotective action of dimethylacetamide and NMA [52]. The in vitro quality assessment of cryopreserved semen did not provide predictive markers of the fertility potential of frozen/thawed sperm in mammalian species as well such as pigs [70] and sheep [71]. In domestic mammals, advanced analytical techniques in the field of genetics and proteomics applied to semen quality assessment have provided new molecular potential markers of sperm freezability. In particular, several sperm and seminal plasma proteins have been identified as potential markers of sperm freezability in the boar, bull, stallion and men [72,73]. The study of the proteomic profile in the sperm of meat-type chickens revealed 50 proteins linked to sperm fertility that are involved in several molecular pathways other than motility, including mitochondria function, sperm maturation, storage within the female reproductive tract and oocyte–sperm interaction [74]. Further studies to better understand sperm biology and fertilization at the molecular level are required to identify novel biomarkers of chicken male fertility to implement cryopreservation technology and improve the long-term in vitro conservation of avian genetic resources. 

## 5. Conclusions

In order to implement sperm cryobanks, semen donors have to be selected to maximize male efficiency and fertility output after the artificial insemination of cryopreserved semen in order to guarantee future progeny. Sperm quality parameters assessed in fresh semen may be related to the in vivo fertilizing ability of cryopreserved semen, and the same relation is lacking if semen quality is assessed after freezing/thawing processing. Therefore, male donors have to be selected according to the quality of fresh ejaculates, irrespective of the sperm outcome after freezing/thawing. Sperm concentration and progressive motility have been successfully used to select male donors with improved fresh semen quality. The frozen/thawed semen of all male donors provided fertile eggs after AI with variable results between breeds and birds within breed. However, conventional sperm quality parameters showed poor predictive potential in the assessment of the fertilizing ability of cryopreserved sperm.

## Figures and Tables

**Table 1:** Descriptive statistics of sperm quality parameters recorded in the fresh ejaculates of *Bionda Piemontese* (BP), *Bianca di Saluzzo* (BS) and *Pepoi* (PP) Italian chicken breeds.

Sperm	BP				BS				PP			
Variables ^1^	Mean	Min	Max	CV	Mean	Min	Max	CV	Mean	Min	Max	CV
VOL (mL)	0.27	0.03	0.83	61.17	0.26	0.03	1.00	70.23	0.35	0.05	0.80	52.88
CONC (×10^9^/mL)	2.49	0.38	5.38	50.21	2.53	0.42	6.31	56.00	4.42	2.33	6.80	21.44
TSO (×10^9^)	0.72	0.03	2.92	84.03	0.71	0.025	2.93	94.13	1.57	0.23	4.3	60.06
SMI (%)	78.50	58.00	98.00	16.16	70.36	44.00	89.00	18.26	92.05	72.00	100.0	6.89
TMS (%)	83.15	62.70	100.0	15.14	74.82	18.10	99.00	39.83	82.06	21.50	99.40	21.90
PMS (%)	18.77	9.90	33.10	40.73	12.73	0.50	25.70	61.87	19.30	2.40	39.20	50.73
VCL (μm/s)	64.80	38.60	131.1	45.27	53.08	25.70	91.10	36.64	56.44	27.40	108.2	32.52
VSL (μm/s)	25.15	16.90	40.30	31.87	19.51	7.80	31.00	31.14	21.67	10.00	39.90	34.14
VAP (μm/s)	39.80	24.50	74.00	38.56	32.52	13.30	54.90	35.61	34.83	17.90	65.90	33.84
LIN (%)	40.96	30.80	53.40	21.02	37.66	30.20	49.80	17.90	38.64	26.60	53.30	15.20
STR (%)	64.57	54.20	75.80	12.59	61.05	54.40	75.10	11.34	62.29	51.40	74.90	8.82
WOB (%)	62.81	56.40	71.40	8.63	61.36	51.60	67.60	7.35	61.63	50.20	70.80	7.27
ALH (μm)	3.76	2.60	5.80	26.42	3.36	1.90	4.40	21.97	3.34	2.10	4.90	15.84
BCF (Hz)	7.06	5.10	9.40	18.58	6.66	5.60	8.40	13.64	7.07	5.20	9.80	12.79

^1^ VOL, sperm volume; CONC, sperm concentration; TSO (VOL*CONC), total sperm output; SMI, sperm membrane integrity; TMS, total motile sperm; PMS, progressive motile sperm; VCL, curvilinear velocity; VSL, straight-line velocity; VAP, average path velocity; LIN (VSL/VCL × 100), linearity; STR (VSL/VAP × 100), straightness; WOB (VAP/VCL × 100), wobble; ALH, amplitude of lateral head displacement; BCF, beat cross frequency.

**Table 2:** Sperm quality variables (LSMeans ± SE) and *p* values in fresh ejaculates of selected semen donors for cryobanking in *Bionda Piemontese* (BP), *Bianca di Saluzzo* (BS) and *Pepoi* (PP) Italian chicken breeds.

Sperm Variables ^1^	Breeds			*p*
	BP	BS	PP	
VOL (mL)	0.43 ± 0.03	0.35 ± 0.03	0.41 ± 0.02	ns
CONC (×10^9^/mL)	3.11 ± 0.17 ^a^	3.67 ± 0.16 ^b^	4.44 ± 0.11 ^c^	0.0001
TSO (×10^9^)	1.34 ± 0.15 ^a^	1.35 ± 0.15 ^a^	1.89 ± 0.10 ^b^	0.0012
SMI (%)	91.59 ± 1.14 ^a^	91.00 ± 1.09 ^a^	95.09 ± 0.77 ^b^	0.0029
TMS (%)	89.52 ± 1.65	90.71 ± 1.57	92.54 ± 1.10	ns
PMS (%)	25.78 ± 1.38 ^a^	24.58 ± 1.32 ^a^	29.90 ± 0.92 ^b^	0.0018
VCL (μm/s)	77.55 ± 3.19 ^a^	76.59 ± 3.05 ^a^	67.36 ± 2.14 ^b^	00.79
VSL (μm/s)	30.02 ± 1.27	28.43 ± 1.21	29.30 ± 0.85	ns
VAP (μm/s)	48.43 ± 2.01	47.05 ± 1.92	44.68 ± 1.35	ns
LIN (%)	39.49 ± 0.82 ^a^	37.37 ± 0.78 ^a^	43.64 ± 0.55 ^b^	0.0001
STR (%)	62.44 ± 0.72 ^a^	60.57 ± 0.69 ^a^	65.65 ± 0.48 ^b^	0.0001
WOB (%)	62.90 ± 0.64 ^a^	61.42 ± 0.61 ^a^	66.23 ± 0.43 ^b^	0.0001
ALH (μm)	3.89 ± 0.08 ^a^	3.92 ± 0.08 ^a^	3.27 ± 0.05 ^b^	0.0001
BCF (Hz)	6.95 ± 0.10 ^a^	6.91 ± 0.09 ^a^	7.74 ± 0.06 ^b^	0.0001

^a,b^. Means within a row with different letters are significantly different. ^1^ VOL, sperm volume; CONC, sperm concentration; TSO (VOL*CONC), total sperm output; SMI, sperm membrane integrity; TMS, total motile sperm; PMS, progressive motile sperm; VCL, curvilinear velocity; VSL, straight-line velocity; VAP, average path velocity; LIN (VSL/VCL × 100), linearity; STR (VSL/VAP × 100), straightness; WOB (VAP/VCL × 100), wobble; ALH, amplitude of lateral head displacement; BCF, beat cross frequency; ns = not significant.

**Table 3:** Sperm quality variables (LSMeans ± SE) and P of the breed factor in frozen/thawed ejaculates of selected donor males for cryobanking in *Bionda Piemontese* (BP), *Bianca di Saluzzo* (BS) and *Pepoi* (PP) Italian chicken breeds.

Sperm Variables ^1^	Breeds			*p*
	BP	BS	PP	
SMI (%)	38.00 ± 3.88	31.60 ± 3.47	31.09 ± 2.39	ns
TMS (%)	31.22 ± 4.53	29.22 ± 4.05	28.28 ± 2.79	ns
PMS (%)	2.91 ± 0.87	3.02 ± 0.78	3.53 ± 0.53	ns
VCL (μm/s)	36.76 ± 1.71	36.77 ± 1.53	36.62 ± 1.06	ns
VSL (μm/s)	11.71 ± 1.24	11.57 ± 1.11	11.98 ± 0.77	ns
VAP (μm/s)	20.21 ± 1.58	20.16 ± 1.41	20.35 ± 0.97	ns
LIN (%)	31.75 ± 2.31	31.02 ± 2.07	31.89 ± 1.43	ns
STR (%)	57.62 ± 2.07	56.75 ± 1.85	57.31 ± 1.27	ns
WOB (%)	54.92 ± 2.29	54.29 ± 2.05	54.78 ± 1.41	ns
ALH (μm)	3.21 ± 0.15 ^a^	2.85 ± 0.13 ^ab^	2.52 ± 0.09 ^b^	0.0014
BCF (Hz)	6.52 ± 0.61	6.18 ± 0.55	6.70 ± 0.38	ns

^a,b^. Means within a row with different letters are significantly different. ^1^ VOL, sperm volume; CONC, sperm concentration; TSO (VOL*CONC), total sperm output; SMI, sperm membrane integrity; TMS, total motile sperm; PMS, progressive motile sperm; VCL, curvilinear velocity; VSL, straight-line velocity; VAP, average path velocity; LIN (VSL/VCL × 100), linearity; STR (VSL/VAP × 100), straightness; WOB (VAP/VCL × 100), wobble; ALH, amplitude of lateral head displacement; BCF, beat cross frequency. ns = not significant.

**Table 4:** Fertility and embryo viability after artificial insemination of frozen/thawed chicken semen of *Bionda Piemontes* (BP), *Bianca di Saluzzo* (BS) and *Pepoi* (PP) Italian breeds.

	Breeds			Fresh	Overall
Parameters	BP	BS	PP	Semen	Value
Fertility ^1^ (%)	4.8 ^a^	15.55	15.89	100 ^a^	16.43 ^b^
Fertile eggs/eggs set (*n*/*n*)	(11/229)	(31/213)	(34/214)	(38/38)	(114/694)
Embryo viability ^2^ (%)	36.36 ^a^	81.25	79.41	86.84	78.26 ^b^
Live embryos/fertile eggs (*n*/*n*)	(4/11)	(25/31)	(27/34)	(33/38)	(89/114)

^a,b^. Different letters within a row indicate a significant difference between the breed and the overall mean value (*p* < 0.001). ^1^ Fertility = (fertile eggs/eggs set) × 100. ^2^ Embryo viability = (live embryos/fertile eggs) × 100.

**Table 5:** Fertility and embryo viability after artificial insemination of frozen/thawed ejaculates in Bionda Piemontese (BP1, BP4 and BP17), Bianca di Saluzzo (BS28, BS35 and BS36) and Pepoi (PP10, PP19 and PP20) Italian chicken breeds.

	BP1	BP4	BP17	BS28	BS35	BS36	PP10	PP19	PP20	Overall Value
Fertility ^1^ (%)	2.7 ^a^	6.98 ^a^	4.35 ^a^	7.5	31 ^a^	8.45	21.74	5.56 ^a^	20.55	16.43 ^b^
Fertile eggs/eggs set (*n*/*n*)	(2/74)	(6/86)	(3/69)	(6/80)	(19/62)	(6/71)	(15/69)	(4/72)	(15/73)	(114/694)
Embryo viability ^2^ (%)	0	66.67	0	66.67	94.74	57.14	73.33	25.00	100	78.26
Live embryos/fertile eggs (*n*/*n*)	(0/2)	(4/6)	(0/3)	(4/6)	(18/19)	(3/6)	(11/15)	(1/4)	(15/15)	(89/114)

^a,b^. Different letters within a row indicate a significant difference between the bird and the overall mean value (*p* < 0.001). ^1^ Fertility = (fertile eggs/eggs set) × 100. ^2^ Embryo viability = (live embryos/fertile eggs) × 100.

## Data Availability

The data presented in this study are available upon request from the corresponding author.

## References

[1] FAO (2020). Domestic Animal Diversity Information System (DAD-IS).

[2] FAO (2007). The State of the World’s Animal Genetic Resources for Food and Agriculture.

[3] FAO Status and Trends of Animal Genetics Resources. Proceedings of the 2022 Intergovernmental Technical Working Group on Animal Genetic Resources for Food and Agriculture, Twelfth Session.

[4] Zanon A., Sabbioni A. (2001). Identificazione e salvaguardia genetica delle razze avicole Italiane. Ann. Med. Vet..

[5] Castillo A., Gariglio M., Franzoni A., Soglia D., Sartore S., Buccioni A., Mannelli F., Cassandro M., Cendron F., Castellini C. (2021). Overview of Native Chicken Breeds in Italy: Conservation Status and Rearing Systems in Use. Animals.

[6] Sabbioni A., Zanon A., Beretti V., Superchi P., Zambini E.M. Carcass yield and meat quality parameters of two Italian autochthonous chicken breeds reared outdoor: Modenese and Romagnolo. Proceedings of the WPSA XII European Poultry Conference.

[7] De Marchi M., Dalvit C., Targhetta C., Cassandro M. (2006). Assessing genetic diversity in indigenous Veneto chicken breeds using AFLP markers. Anim. Genet..

[8] Rizzi C., Marangon A. (2012). Quality of organic eggs of hybrid and Italian breed hens. Poult. Sci..

[9] Zanetti E., De Marchi M., Dalvit C., Cassandro M. (2010). Genetic characterization of local Italian breeds of chickens undergoing in situ conservation. Poult. Sci..

[10] Madeddu M., Zaniboni L., Mangiagalli M.G., Cassinelli C., Cerolini S. (2013). Egg related parameters affecting fertility and hatchability in the Italian bantam breed Mericanel della Brianza. Anim. Reprod. Sci..

[11] Cozzi M.C., Colombo E., Zaniboni L., Madeddu M., Mosca F., Strillacci M.G., Longeri M., Bagnato A., Cerolini S. (2017). Phenotypic and genetic characterization of the Italian bantam chicken breed Mericanel della Brianza. Livest. Sci..

[12] Mosca F., Zaniboni L., Stella S., Kuster C.A., Iaffaldano N., Cerolini S. (2018). Slaughter performance and meat quality of Milanino chickens reared according to a specific free-range program. Poult. Sci..

[13] Gualtieri M., Pignattelli P., Cristalli A. (2006). Pollo di razza valdarnese bianca. Risorse Genetiche Animali Autoctone Della Toscana.

[14] MIPAAF (2014). Disciplinare del Registro Anagrafico Degli Avicoli Autoctoni.

[15] Anci-Aia, Razze Avicole Autoctone. www.anci-aia.it.

[16] Gandini G., Oldenbroek K., Oldenbroek K. (2007). Strategies for moving from conservation to utilisation. Chapter 2 in Utilisation and Conservation of Farm Animal Genetic Resources.

[17] Sun Y., Yunlei L., Zong Y., Mehaisen G.M.K., Chen J. (2022). Poultry genetic heritage cryopreservation and reconstruction: Advancement and future challenges. J. Anim. Sci. Biotechnol..

[18] Mazur P., Johnson L.A., Larsson K. (1985). Basic concepts in freezing cells. Proceedings of the First International Conference on Deep Freezing of Boar Semen.

[19] Meuwissen T.H.E., Oldenbroek J.K. (1999). Operation of conservation schemes. Genebanks and the Conservation of farm Animal Genetic Resources.

[20] Blesbois E. (2012). Biological features of the avian male gamete and their application to biotechnology of conservation. J. Poult. Sci..

[21] Smith C., FAO (1984). Genetic aspects of conservation in farm livestock. Animal Genetic Resources Conservation by Management, Data Banks and Training, Proceedings of the Joint FAO/UNEP Expert Panel Meeting, October 1983 Part 1.

[22] FAO (2012). Cryoconservation of animal genetic resources. FAO Animal Production and Health Guidelines No. 12.

[23] Lake P. (1983). Factors affecting the fertility level in poultry, with special references to artificial insemination. World’s Poultry Sci. J..

[24] Mohan J., Sharma S.K., Kolluri G., Dhama K. (2018). History if artificial insemination in poultry, its components and significance. World’s Poult. Sci. J..

[25] Bakst M.R., Dymond J.S., Lemma A. (2013). Artificial insemination in poultry. Success in Artificial Insemination—Quality of Semen and Diagnostics Employed.

[26] Sauveurs B., Sauveur B., De Reviers M. (1988). Reproduction naturelle et insemination artificielle. Reproduction des Volailles et Production D’oeufs.

[27] Iaffaldano N., Di Iorio M., Rusco G., Antenucci E., Zaniboni L., Madeddu M., Marelli S., Schiavone A., Soglia D., Buccioni A. (2021). Italian semen cryobank of autochthonous chicken and turkey breeds: A tool for preserving genetic biodiversity. Ital. J. Anim. Sci..

[28] (2010). Fass, Guide for the Care and Use of Agricultural Animals in Research and Teaching.

[29] Burrows W.H., Quinn J.P. (1935). A method of obtaining spermatozoa from the domestic fowl. Poult. Sci..

[30] Brillard J.P., McDaniel G.R. (1985). The reliability and efficiency of various methods for estimating spermatozoa concentration. Br. Poult. Sci..

[31] Mosca F., Madeddu M., Sayed A.A., Zaniboni L., Iaffaldano N., Cerolini S. (2016). Combined effect of permeant and non-permeant cryoprotectants on the quality of frozen/thawed chicken sperm. Cryobiology.

[32] Rosato M.P., Iaffaldano N. (2011). Effect of chilling temperature on the long-term survival of rabbit spermatozoa held either in a Tris based or a jellified extender. Reprod. Domest. Anim..

[33] Lake P.E., Ravie O. (1979). Effect on fertility of storing fowl semen for 14 h at 5 °C in fluids of different pH. J. Reprod. Fertil..

[34] Zaniboni L., Madeddu M., Mosca M., Ahmad A.S., Marelli S.P., Di Iorio M., Iaffaldano N., Cerolini S. (2022). Concentration dependent effect of dimethylacetamide and N-methylacetamide on the quality and fertility of cryopreserved chicken semen. Cryobiology.

[35] Madeddu M., Mosca F., Abdel Sayed A., Zaniboni L., Mangiagalli M.G., Colombo E., Cerolini S. (2016). Effect of cooling rate on the survival of cryopreserved rooster sperm: Comparison of different distances in the vapour above the surface of the liquid nitrogen. Anim. Reprod. Sci..

[36] Burrows W.H., Quinn J.P. (1939). Artificial Insemination of Chickens and Turkeys.

[37] SAS (1999). SAS, User’s Guide Statistics.

[38] Hammer Ø., Harper D.A.T., Ryan P.D. (2001). PAST: Paleontological statistics software package for education and data analysis. Palaeontol. Electron..

[39] Bortoluzzi C., Crooijmans R.P.M.A., Bosse M., Hiemstra S.J., Groenen M.A., Megens H.J. (2018). The effects of recent changes in breeding preferences on maintaining traditional Dutch chicken genomic diversity. Heredity.

[40] Mastrangelo S., Ciani E., Sardinia M.T., Sottile G., Pilla F., Portolano B., Bi O.V. (2018). Ita Consortium. Runs of homozygosity reveal genome-wide autozygosity in Italian sheep breeds. Anim. Genet..

[41] Malomane D.K., Simianer H., Weigend A., Reimer C., Schmitt A.O., Weigend S. (2019). The SYNBREED chicken diversity panel: A global resource to assess chicken diversity at high genomic resolution. BMC Genom..

[42] Blesbois E., Seigneurin F., Grasseau I., Limouzin C., Besnard J., Gourichon D., Coquerelle G., Rault P., Tixier-Boichard M. (2007). Semen cryopreservation for *ex situ* management of genetic diversity in chicken: Creation of the French avian cryobank. Poult. Sci..

[43] Santiago-Moreno J., López-Sebastián A., Castaño C., Coloma M.A., Gómez-Bruneta A., Toledano-Díaz A., Prieto M.T., Campo J.L. (2009). Sperm variables as predictors of fertility in Black Castellana roosters: Use in the selection of sperm donors for genome resources banking purposes. Span. J. Agric. Res..

[44] Mussa N.J., Boonkum W., Chankitisakul V. (2023). Semen quality traits of two native Thai native chickens producing a high and a low of semen volumes. Vet. Sci..

[45] Kowalczyk A., Łukaszewicz E. (2015). Simple and Effective Methods of Freezing Capercaillie (*Tetrao urogallus* L.) *Semen*. PLoS ONE.

[46] Froman D.P., Feltmann A.J. (1998). Sperm mobility: A quantitative trait of the domestic fowl (*Gallus domesticus*). Biol. Reprod..

[47] Froman D.P., Feltman A.J. (2000). Sperm mobility: Phenotype in roosters (*Gallus domesticus*) determined by concentration of motile sperm and straight line velocity. Biol. Reprod..

[48] Siudzinska A., Lukaszewicz E. (2008). The effect of breed on freezability of semen of fancy fowl. Anim. Sci. Pap. Rep..

[49] Partika A., Lukaszewicz E., Nizanski W. (2012). Effect of cryopreservation on sperm parameters, lipid peroxidation and antioxidant enzymes activity in fowl semen. Theriogenology.

[50] Makhafola M.B., Lehloenya K.C., Mphaphthi M.L., Dinnyes A., Nedambale T.L. (2009). The effect of breed on the survivability and motility rate of cryopreserved cock semen. S. Afr. J. Anim. Sci..

[51] Mosca F., Madeddu M., Sayed A.A., Zaniboni L., Iaffaldano N., Cerolini S. (2016). Data on the positive synergic action of dimethylacetamide and trehalose on quality of cryopreserved chicken sperm. Data Brief.

[52] Mosca F., Zaniboni L., Abdel Sayed A., Madeddu M., Iaffaldano N., Cerolini S. (2019). Effect of dimethylacetamide and N-methylacetamide on the quality and fertility of frozen/thawed chicken semen. Poult. Sci..

[53] Tselutin K., Seigneurin F., Blesbois E. (1999). Comparison of cryoprotectants and methods of cryopreservation of fowl spermatozoa. Poult. Sci..

[54] Long J.A. (2006). Avian Semen Cryopreservation: What Are the Biological Challenges?. Poult. Sci..

[55] Long J.A., Bongalhardo D.C., Pelaéz J., Saxena S., Settar P., O’Sullivan N.P., Fulton J.E. (2010). Rooster semen cryopreservation: Effect of pedigree line and male age on postthaw sperm function. Poult. Sci..

[56] Janosikova M., Petricakova K., Ptacek M., Savvulidi F.G., Rychtarova J., Fulka J. (2023). New approaches for long-term conservation of rooster spermatozoa. Physiol. Reprod..

[57] Shanmugan M., Pranay Kumar K., Mahapatra R.K., Anand N.L. (2018). Effect of different cryoprotectants on post-thaw semen parameters and fertility in Nicobari chickens. Indian J. Poult. Sci..

[58] Pranay Kumar K., Swathi B., Shanmugam M. (2018). Cryopreservation of rooster semen using N-methylacetamide as cryoprotective agent. Int. J. Agric. Sci..

[59] Kim S.W., Choi J.S., Ko Y.G., Do Y.J., Byun M., Park S.B., Seong H.H., Kim C.D. (2014). Effect of N-methylacetamide concentration on the fertility and hatchability of cryopreserved Ogye rooster semen. Korean J. Poult. Sci..

[60] Lee H.J., Kim S.K., Jang H.J., Kang K.S., Kim J.H., Choi S.B., Han J.Y. (2012). Cryopreservation of Korean Oge chicken semen using N-methylacetamide. Cryoletters.

[61] Sasaki K., Tatsumi T., Tsutsui M., Niinomi T., Imai T., Naito M., Tajima A., Nishi Y. (2010). A method for cryopreserving semen from Yakido roosters using N-methylacetamide as a cryoprotective agent. J. Poult. Sci..

[62] Abouelezz F.M.K., Castaño C., Toledano-Díaz A., Esteso M.C., Lopez-Sebastián A., Campo J.L., Santiago-Moreno J. (2015). Effect of the interaction between cryopotectant concentration and cryopreservation method on frozen/thawed chicken sperm variables. Reprod. Domest. Anim..

[63] Abouelezz F.M.K., Sayed M.A.M., Santiago-Moreno J. (2017). Fertility disturbances of dimethylacetamide and glycerol in rooster sperm diluents: Discrimination among effects produced pre and post freezing-thawing process. Anim. Reprod. Sci..

[64] Thélie A., Bailliard A., Seigneurin F., Zerjal T., Tixier-Boichard M., Blesbois E. (2019). Chicken semen cryopreservation and use for the restoration of rare genetic resources. Poult. Sci..

[65] Donoghue A.M., Wishart G.J. (2000). Storage of poultry semen. Anim. Reprod. Sci..

[66] Blesbois E. (2011). Freezing avian semen. Avian Biol. Rev..

[67] Blesbois E., Grasseau I., Seigneurin F., Mignon-Grasteau S., Saint Jalme M., Mialon-Richard M.M. (2008). Predictors of success of semen cryopreservation in chickens. Theriogenology.

[68] Kowalczyk A., Łukaszewicz E., Rzońca Z. (2012). Successful preservation of capercaillie (*Tetrao urogallus* L.) semen in liquid and frozen states. Theriogenology.

[69] Łukaszewicz E., Kowalczyk A., Rzońca Z. (2011). Characteristics of fresh semen of captive-bred capercaillie *Tetrao urogallus* L.. Zoo Biol..

[70] Sellés E., Gadea J., Romar R., Matas C., Ruiz S. (2003). Analysis of in vitro fertilizing capacity to evaluate the freezing procedures of boar semen and to predict the subsequent fertility. Reprod. Domest. Anim..

[71] Papadopoulos S., Hanrahan J.P., Donovan A., Duffy P., Boland M.P., Lonergan P. (2005). In vitro fertilization as a predictor of fertility from cervical insemination of sheep. Theriogenology.

[72] Yànez-Ortiz I., Catalàn J., Rodríguez-Gil J.E., Miró J., Yeste M. (2022). Advances in sperm cryopreservation in farm mammals: Cattle, horse, pig and sheep. Anim. Reprod. Sci..

[73] Duracka M., Benko F., Tvrdá E. (2023). Molecular markers: A new paradigm in the prediction of sperm freezability. Int. J. Mol. Sci..

[74] Carvalho A.V., Soler L., Thélie A., Grasseau I., Cordeiro L., Tomas D., Teixeira-Gomes A.P., Labas V., Blesbois E. (2021). Proteomic changes associated with sperm fertilizing ability in meat-type roosters. Front. Cell Dev. Biol..

